# ^18^F-sodium fluoride positron emission tomography assessed microcalcifications in culprit and non-culprit human carotid plaques

**DOI:** 10.1007/s12350-018-1325-5

**Published:** 2018-06-25

**Authors:** H. Hop, S. A. de Boer, M. Reijrink, P. W. Kamphuisen, M. H. de Borst, R. A. Pol, C. J. Zeebregts, J. L. Hillebrands, R. H. J. A. Slart, H. H. Boersma, J. Doorduin, D. J. Mulder

**Affiliations:** 1 Division of Vascular Medicine, Department of Internal Medicine, University Medical Center Groningen, University of Groningen, Hanzeplein 1, 9713 GZ Groningen, The Netherlands; 2 Division of Nephrology, Department of Internal Medicine, University Medical Center Groningen, University of Groningen, Groningen, The Netherlands; 3 Division of Vascular Surgery, Department of Surgery, University Medical Center Groningen, University of Groningen, Groningen, The Netherlands; 4 Division of Pathology, Department of Pathology and Medical Biology, University Medical Center Groningen, University of Groningen, Groningen, The Netherlands; 5 Department of Nuclear Medicine and Molecular Imaging, University Medical Center Groningen, University of Groningen, Groningen, The Netherlands; 6 Department of Biomedical Photonic Imaging, University of Twente, Enschede, The Netherlands; 7 Department of Clinical Pharmacy and Pharmacology, University Medical Center Groningen, University of Groningen, Groningen, The Netherlands

**Keywords:** ^18^F-sodium fluoride (^18^F-NaF), Calcification, PET/CT imaging, Vulnerable atherosclerotic plaque, Carotid artery stenosis

## Abstract

**Background:**

^18^F-NaF positron emission tomography (PET) targets microcalcifications. We compared in vitro microPET assessed ^18^F-NaF uptake between culprit and non-culprit human carotid plaques. Furthermore, we compared ^18^F-NaF uptake with calcification visualized on microcomputed tomography (microCT).

**Methods:**

Carotid plaques from stroke patients undergoing surgery were incubated in ^18^F-NaF and scanned using a microPET and a microCT scan. The average PET assessed ^18^F-NaF uptake was expressed as percentage of the incubation dose per gram (%Inc/g). ^18^F-NaF PET volume of interest (VOI) was compared with CT calcification VOI.

**Results:**

23 carotid plaques (17 culprit, 6 non-culprit) were included. The average ^18^F-NaF uptake in culprit carotid plaques was comparable with the uptake in non-culprit carotid plaques (median 2.32 %Inc/g [IQR 1.98 to 2.81] vs. median 2.35 %Inc/g [IQR 1.77 to 3.00], *P *= 0.916). Only a median of 10% (IQR 4 to 25) of CT calcification VOI showed increased ^18^F-NaF uptake, while merely a median of 35% (IQR 6 to 42) of ^18^F-NaF PET VOI showed calcification on CT.

**Conclusions:**

^18^F-NaF PET represents a different stage in the calcification process than CT. We observed a similar PET assessed ^18^F-NaF uptake and pattern in culprit and non-culprit plaques of high-risk patients, indicating that this method may be of more value in early atherosclerotic stenosis development.

**Electronic supplementary material:**

The online version of this article (10.1007/s12350-018-1325-5) contains supplementary material, which is available to authorized users.

## Spanish Abstract

**Antecedentes:**

La tomografía por emisión de positrones (PET) con ^18^F-floruro de sodio (^18^F-NaF) demuestra microcalcificaciones. En este estudio, comparamos la captación de ^18^F-NaF entre placas carotideas culpables y no culpables valorada con microPET *in vitro.* Además, comparamos la captación de ^18^F-NAF con las calcificaciones detectadas por microtomografía computada (microCT).

**Métodos:**

Placas carotídeas de pacientes postoperados que sufrieron de un evento vascular cerebral fueron incubadas en 18-NaF y escaneadas usando microPET y microCT. El promedio de la captación fue expresado como porcentaje de la dosis de incubación por gramo (%Inc/gr). El volumen de interés (VOI) del PET ^18^F-NaF se comparó con el VOI de calcificación por CT.

**Resultados:**

**Se incluyeron** 23 placas carotídeas (17 culpables y 6 no culpables). El promedio de la captación de ^18^F-NaF en placas carotídeas culpable fue comparable al de las placas no culpables. (mediana 2.32%Inc/gr [IQR1.98-2.81] vs. mediana 2.35%Inc/gr [IQR1.77-3], p=.916). Solo una mediana de 10% (IQR 4-25) del VOI en CT mostró un aumento en la captación de ^18^F-NaF, mientras una mediana de 35% (IQR 6-42) del VOI de PET ^18^F-NaF mostró calcificación en CT.

**Conclusiones:**

La tomografía por emisión de positrones con ^18^F-NaF evalúa una etapa distinta en el proceso de calcificación que la CT. El patrón de captación de ^18^F-NaF que observamos en este estudio fue similar entre placas culpables y no culpables de pacientes de alto riesgo, lo cual indica que este método puede ser de mayor valor en el desarrollo temprano de la estenosis ateroesclerótica.

## Chinese Abstract

**背景:**

F18 氟化钠正电子发射断层扫描 (PET) 可用于检测微钙化。我们使用微型 PET 来评估病人体外 F18 氟化钠在颈动脉罪犯和非罪犯斑块之间的的摄取区别。此外, 我们还将 F18 氟化钠的摄取与 microCT 扫描检测的钙化进行对比。

**方法:**

将接受手术的中风患者的颈动脉斑块在 F18 氟化钠中培养并用 microPE 和 microCT 进行扫描。PET 对 F18 氟化钠的平均摄取率表示为每克培养剂量的百分比。F18 氟化钠 PET 中的感兴趣区域和 CT 钙化图像中的感兴趣区域 (VOI) 进行比较。

**结果:**

一共有 23个颈动脉斑块, 其中 17个罪犯斑块和 6个非罪犯斑块。罪犯和非罪犯颈动脉斑块的 F18 氟化钠的平均摄取率无明显差异 (中值 2.32 % Inc/g [IQR 1.98-2.81] vs. 中值2.35 % Inc/g [IQR 1.77-3.00], p = 0.916)。仅中值为10% [IQR 4-25] 的 CT 钙化 VOI 显示有F18氟化钠的摄取增加, 中值为 35%[IQR 6-42] 的 F18 氟化钠 PET VOI 显示有CT 上的钙化。

**结论:**

相较于 CT, F18 氟化钠 PET 展现了钙化过程中的一个不同阶段。在高危病人中, 我们观察到罪犯和非罪犯斑块具有类似的 PET F18 氟化钠摄取和模式, 表明该方法可能在早期动脉粥样硬化狭窄发展中具有更高的价值。

## French Abstract

**Contexte:**

La tomographie par émission de positons (TEP) au ^18^F-NaF marque les micro-calcifications. Nous avons comparé in vitro les plaques carotidiennes humaines responsable d’accident vasculaire cérébrales marquées au ^18^F-NaF avec une microTEP et leur calcification au moyen d’un microCT

**Méthodes:**

Les plaques carotidiennes de patients victimes d’un accident vasculaire cérébral qui ont bénéficié d’ une intervention chirurgicale ont été incubées dans du ^18^F-NaF et imagées avec une microTEP et un microCT. Le pourcentage d’absorption moyen de ^18^F-NaF par gr de tissu (% Inc/g) ont été comparées au volume d’intérêt (VOI) des calcifications mesurées par CT.

**Résultats:**

23 plaques carotidiennes (17 responsables d’un AVC et 6 non responsables) ont été incluses. Le % moyen d’absorption de ^18^F-NaF dans les plaques carotidiennes responsables furent comparable à l’absorption dans les plaques non responsables (2,32% Inc/g vs 2,35% Inc/g , p = 0,916). Dix pourcent seulement des calcifications visualisées par CT démontrèrent une augmentation d’absorption de ^18^F-NaF et 35% des plaques montrant une absorption de ^18^F-NaF PET VOI démontrèrent des calcifications au CT.

**Conclusions:**

L’imagerie de l’absorption de ^18^F-NaF par tomographie à positrons représente un processus physiopathologique différent de l’imagerie des calcifications par CT. L’absorption du ^18^F-NaF par les plaques responsables et non responsables d’accidents vasculaire cérébraux pourrait s’avérer utile dans le diagnostic précoce des sténoses athéromateuses.

## Introduction

Surgical removal of atherosclerotic plaques from the carotid artery highly reduces the risk of future stroke in symptomatic patients with ≥ 70% stenosis.^1^ However, most of these patients will not have a new event when treated with best medical therapy.^2^ Furthermore, the role of surgery in moderate symptomatic stenosis (50% to 69%) and asymptomatic stenosis is under debate.^3^–^5^ Therefore, taking into account the potential risk for surgical complications, the selection of patients who will benefit most from surgery is challenging.

In order to improve risk stratification, research has been focused on the identification of plaques at risk for rupture, so-called vulnerable plaques.^6^–^8^ Currently, plaque thickness and intraplaque processes, such as inflammation and microcalcification, are seen as important contributors to vulnerability. These processes have become targets of various molecular imaging techniques, as they potentially allow non-invasive risk stratification of individual patients with carotid artery stenosis.^9^,^10^

Recently, several studies have shown the feasibility of ^18^F-sodium fluoride (^18^F-NaF) positron emission tomography (PET) for imaging of atherosclerotic plaques.^11^–^13^^18^F-NaF predominantly binds to areas of microcalcification within the plaque.^14^ Appearance of microcalcifications indicates the active formation of calcification and is associated with plaque vulnerability.^15^,^16^ In contrast, established calcifications are seen as atherosclerotic end stage products and are associated with plaque stability.^17^–^20^

It has been suggested that ^18^F-NaF may additionally be a useful marker for plaque vulnerability.^21^ Indeed, a clinical study by Joshi et al. showed that ruptured and high-risk coronary plaques have a significantly higher ^18^F-NaF uptake than non-culprit and low-risk coronary plaques.^22^ However, data on ^18^F-NaF uptake in carotid plaques are limited and their usefulness for the prediction of future stroke is unclear.^23^–^25^ Additionally, limited data have been published on the relation between active microcalcifications and established calcifications in human carotid plaques.^26^,^27^

The primary objective of this study is to compare in vitro microPET assessed ^18^F-NaF uptake between culprit and non-culprit carotid plaques from stroke patients, using non-macrocalcified renal arteries from healthy kidney donors as negative controls. The secondary objective is to compare the distribution of ^18^F-NaF uptake on microPET with calcification visualized on a high-resolution microcomputed tomography (microCT) in carotid plaques.

## Materials and Methods

### Study Subjects

Carotid plaques were collected from stroke patients who underwent carotid endarterectomy (CEA) at the Department of Surgery (Division of Vascular Surgery) of the University Medical Center Groningen (UMCG), between July 2015 and March 2016. Indication for CEA was decided by a surgeon expert panel and was based on the presence of symptomatic (culprit) stenosis (≥ 50%) or asymptomatic (non-culprit) stenosis (≥ 70%) of the internal carotid artery, according to internal guidelines.^28^,^29^ One patient with < 50% stenosis was selected for CEA because of an irregular aspect of the plaque surface.

In order to increase the reliability of our measurements, we used renal artery specimens from healthy kidney donors as negative controls. The specimens were obtained during living donor nephrectomy.

Clinical and demographic data from the included patients were collected from medical records. In the group with culprit plaques, medication use and history of cardiovascular diseases prior to the recent event were registered. The study was reviewed by the ethics committee of the UMCG (METc 2015/258). All patients gave written informed consent.

### Study Procedure

Immediately after excision, carotid plaques and renal artery specimens were placed into phosphate buffered saline (PBS) and kept on ice. Both were incubated for one hour in 49.4 ± 7.2 MBq ^18^F-NaF in 20 mL. After incubation, the plaques and renal arteries were carefully rinsed 5 times with 10 mL PBS. Then, tissue samples were weighed and microPET and microCT scans were performed. After the imaging procedure, the carotid plaques were cut transversely into segments of 3 to 4 mL. The renal arteries had a maximum thickness of 5 mL and therefore no cross-sections were made. The segments were embedded in paraffin for histological analysis.

### Production of ^18^F- NaF

^18^F-NaF was produced by passing a solution of ^18^F-fluoride in water over a quaternary methyl ammonium (QMA) light anion exchange cartridge (Waters Chromatography B.V., Etten-Leur, The Netherlands). After washing the QMA with water, [^18^F]-fluoride was eluted with saline and passed over a sterile Millex GS 0.22 µm filter (Millipore B.V., Amsterdam, The Netherlands). The radiochemical purity for all runs was > 95%.

### PET and CT Acquisition

Carotid plaques and renal arteries were positioned into a microPET scanner (MicroPET Focus 220, Siemens Medical Solutions USA, Knoxville, TN, USA), and an emission scan of 30 minutes was performed. After the PET scan was finished, the bed of the PET scanner was transferred to a microCT scanner (Inveon CT, Siemens Medical Solutions USA, Knoxville, TN, USA) without moving or touching the tissue samples. The CT exposure settings were 50 keV and 500 µAs, and a 100-ms exposure time for 360 projections during one 360° rotation.

The PET scans were reconstructed into a single frame of 30 minutes, using OSEM2D (4 iterations and 16 subsets), after being normalized and corrected for attenuation and decay of radioactivity. The CT images were reconstructed with the Feldkamp algorithm.^30^

### Data Analysis

The PET and CT images were automatically registered using PMOD 3.7 (PMOD Technologies LLC, Zürich, Switzerland). The registration was visually inspected and manually corrected when necessary. For quantification of the average ^18^F-NaF uptake, three-dimensional volumes of interest (VOIs) were drawn around the whole tissue samples. The uptake (in kBq/cc) was corrected for weight of the specimen and the incubation dose, and expressed as percentage uptake of total incubation dose per gram of tissue (%Inc/g). It was assumed that 1 cubic centimeter equals 1 gram of tissue.

VOIs were also automatically drawn around ^18^F-NaF PET areas with a threshold of ≥ 50% of the maximum ^18^F-NaF uptake and assigned as ^18^F-NaF PET VOI. VOIs were automatically drawn around CT areas with a Hounsfield Unit (HU) ≥ 1000 and assigned as CT calcification VOI. The threshold of 50% of the maximum uptake value was chosen in order to select the volume with the highest ^18^F-NaF uptake, and thereby minimize the bias of a partial volume effect.^27^ The HU of 1000 was based on the CT scan of a phantom with various known calcium hydroxyapatite densities, whereby a lower threshold was chosen in order to not miss any calcification. To determine the overlap between the ^18^F-NaF PET VOIs and CT calcification VOIs, an intersection VOI was automatically drawn. Then, the CT calcification area (HU ≥ 1000) within the ^18^F-NaF PET VOI was measured and expressed as a percentage of the ^18^F-NaF PET VOI; and the other way around; ^18^F-NaF PET uptake area (≥ 50% of maximum ^18^F-NaF uptake) within the CT calcification VOI was measured and expressed as a percentage of the CT calcification VOI.

### Histological Staining

To validate our data, von Kossa and alizarin red stainings for calcification were performed on two plaque segments without any CT calcification, but with clear ^18^F-NaF uptake. The pattern of ^18^F-NaF uptake on PET images was compared with the results of histology. Furthermore, the two renal arteries with the highest ^18^F-NaF uptake were selected for staining (negative controls). Only negligible ^18^F-NaF uptake was expected and no calcification in the renal arteries, because only healthy kidney donors with a renal vasculature without any signs of atherosclerosis are eligible for transplantation.

To obtain further information on the vulnerability of included carotid plaques, segments of five culprit and three non-culprit plaques were assessed for histological features of vulnerability. The segments were stained with Martius Scarlet Blue (MSB) using histochemistry and for CD68- and CD34-expressing cells using immunohistochemistry.^31^ With these markers, the presence of intraplaque thrombus and collagenous fibrous cap (MSB), inflammation (CD68-positive macrophages), and intraplaque microvessels (CD34-positive endothelial cells) could be identified. One observer, highly experienced in vascular pathology, visual inspected and interpreted the histological features, and compared the culprit and non-culprit plaques. For a detailed description of the staining procedures, see Online Resource 1.

### Statistical Analysis

Descriptive data are presented as frequencies (percentage), median (interquartile range), or mean ± SD. Based on the distribution of data (tested by normal probability plots), differences between data were analyzed with non-parametric tests. For continuous data the Mann–Whitney *U* test (two groups) or the Kruskal–Wallis test (≥ two groups) was used. Categorical data were analyzed with the Chi Square test. A Spearman Correlation was used to test the association between continuous data. A two-sided *P*-value ˂ 0.05 was considered statistically significant. Statistical analyses were performed using SPSS for Windows (version 23.0).

## Results

### Patient Characteristics

We included 23 carotid plaques (17 culprit and 6 non-culprit) from 23 patients (median age 72 years, interquartile range [IQR] 61 to 75, 85% male) who had undergone CEA, and 15 renal artery specimen from healthy kidney donors (Table 1). The demographic and clinical characteristics were comparable between stroke patients with culprit and non-culprit plaques (Table 2). Only BMI was higher in the non-culprit group (*P *= 0.020). The mean time between the cerebrovascular event (stroke, TIA or amaurosis fugax) and CEA in the group with culprit plaques was 21 ± 14 days. All patients in the non-culprit group had a history of stroke related to the contralateral carotid artery. The time range between that stroke and the recent non-culprit CEA was 2 to 23 months. The healthy kidney donors (from whom renal artery segments were obtained) were younger than CEA patients (*P *= 0.001) and had no history of cardiovascular disease.

**Table 1:** Included specimens

	Culprit plaque	Non-culprit plaque	Renal artery specimen
Number included	17	6	15
Number PET performed	17	6	15
Number CT performed	15	1	8

**Table 2:** Clinical characteristics

	Culprit plaques (n=17)	Non-culprit plaques (n=6)	Renal arteries (n=15)
Age (years)	72 (64–76)	71 (55–72)	55 (41–63)
Sex, male	14 (82)	5 (83)	5 (33)
Stenosis degree (%)			
70–99	14 (82)	6 (100)	–
50–69	2 (12)	–	–
< 50	1 (6)	–	–
Presenting symptoms			
Stroke	9 (53)	–	–
TIA	7 (41)	–	–
Amaurosis fugax	1 (6)	–	–
Cardiovascular history	8 (47)	6 (100)	0
Coronary artery disease	3 (18)	3 (50)	–
Cerebrovascular disease^a^	4 (24)	6 (100)	–
Peripheral artery disease	4 (24)	2 (33)	–
Diabetes mellitus	1 (6)	3 (50)	0
Current smoker	6 (34)	2 (33)	6 (40)
BMI (kg/m^2^)	25 (23–30)	31 (29–31)	26 (24–28)
SBP (mm Hg)	139 (132–150)	144 (127–173)	134 (127–148)
DBP (mm Hg)	76 (60–83)	73 (68–78)	76 (70–83)
Total cholesterol (mmol/L)	4.4 (3.5–6.1)	4.2 (3.4–10)	5.5 (4.8–6.2)
LDL cholesterol (mmol/L)	3 (2.2–3.8)	2.5 (2.0–8.2)	3.1 (2.7–4.1)
Medication^b^			
Antihypertensives	9 (53)	3 (50)	3 (20)
Statins	7 (41)	6 (100)	1 (6)
Antiplatelet therapy	5 (29)	6 (100)	0
Anticoagulation	3 (18)	0	0

Data are expressed as number (%) or median (interquartile range)

*TIA*, transient ischemic attack; *BMI*, body mass index; *SBP*, systolic blood pressure; *DBP*, diastolic blood pressure; *LDL*, low-density lipoprotein

^a^In culprit plaques: other than current event

^b^In culprit plaques: medication use prior to the recent cerebrovascular event

### Visual Assessment of PET and CT Images

**Figure 1:**
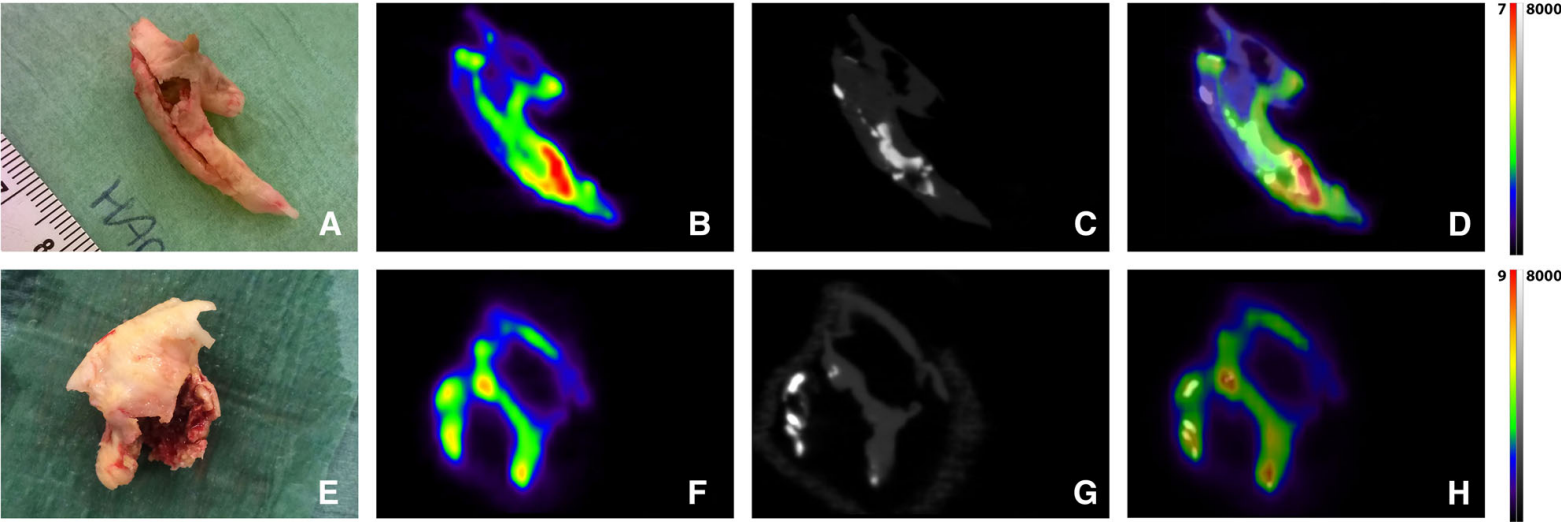
^18^F-NaF microPET and microCT images of human carotid plaque. (**A**, **E**) Human carotid plaque after carotid endarterectomy. (**B**, **F**) Sagittal view of *ex vivo* PET showing a heterogeneous distribution of ^18^F-NaF uptake with a clear hotspot (red/yellow). (**C**, **G**) Sagittal view of corresponding microCT images. On the background of example G are the contours of the CT bed visible. (**D**, **H**) Fused images showing different distributions of microcalcification (^18^F-NaF PET) and established calcification (microCT). *Scale PET images in %Inc/g, scale CT images in HU*

**Figure 2:**
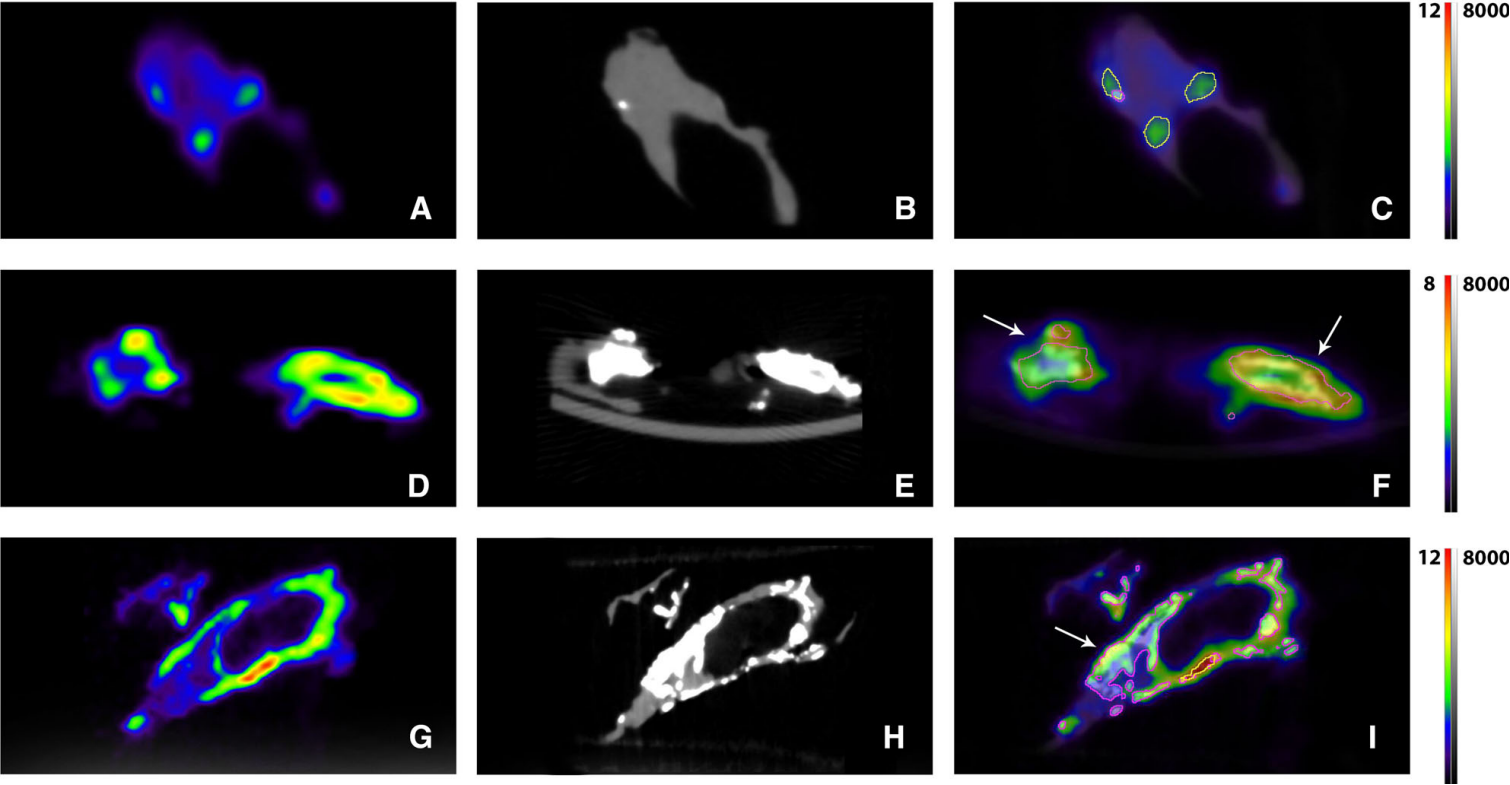
Sagittal views of ^18^F-NaF microPET and microCT of human carotid plaques. (**A**, **D**, **G**) PET areas with ^18^F-NaF uptake. (**B**, **E**, **H**) CT images showing calcification. In image B only one small calcification can be seen in the areas of ^18^F-NaF uptake. (**C**, **F**, **I**) Fused images. Volumes of interest are drawn around CT calcification areas (white calcification, pink line). The relatively large calcifications (white arrows) show the most intense ^18^F-NaF uptake at their surface. In the smaller calcifications this cannot be observed due to the limited resolution. *Scale PET images in %Inc/g, scale CT images in HU*

PET images of all 23 plaques, showed a heterogeneous ^18^F-NaF uptake distribution and clear hotspots (Figure 1). The registered PET and CT images (n = 16) showed a discordant pattern between CT assessed calcification and ^18^F-NaF PET assessed calcification. In all plaques, ^18^F-NaF uptake was seen in regions without calcifications visualized on CT scan. In the largest CT calcification volumes ^18^F-NaF uptake was only seen at the outer surface (Figure 2). The ^18^F-NaF uptake in the renal arteries was only visible when a low ^18^F-NaF uptake threshold was chosen compared with the carotid plaques, and no calcification was visible on the CT (n = 8).

## ^18^F-NaF Uptake in Culprit and Non-culprit Plaques

**Figure 3:**
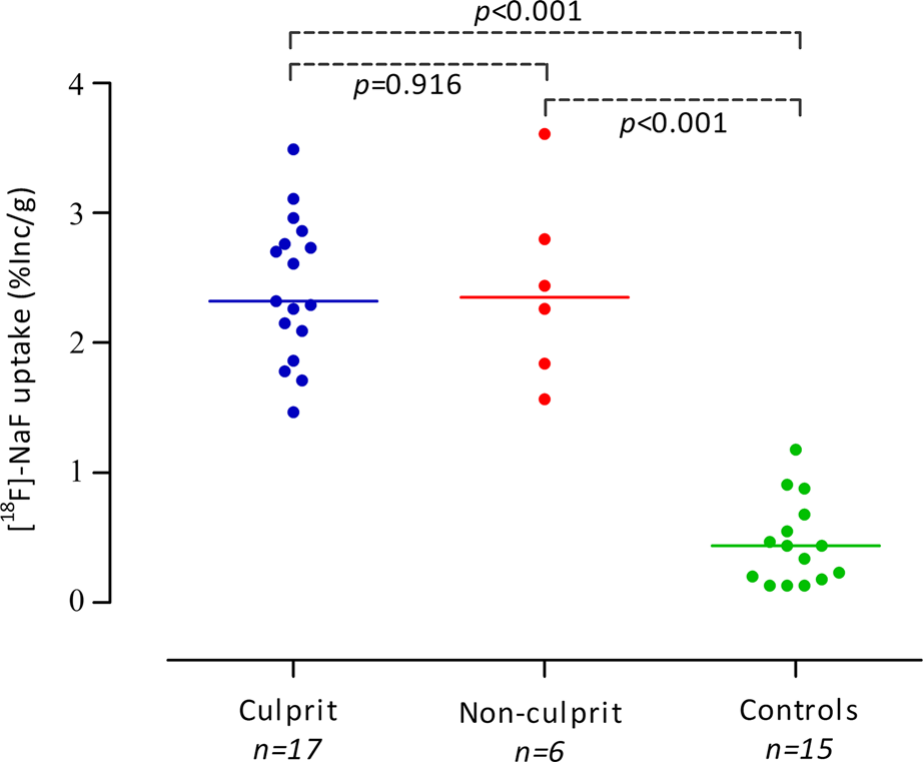
^18^F-NaF uptake, as measure of microcalcification, in human carotid plaques and controls. PET assessed ^18^F-NaF uptake in culprit and non-culprit carotid plaques. Renal arteries of healthy kidney donors are used as negative controls. Data is expressed as percentage ^18^F-NaF uptake of the total incubation dose per gram of tissue (% Inc/g). The horizontal line represents the median

The average ^18^F-NaF uptake was similar in culprit and non-culprit carotid plaques (median 2.32 %Inc/g [IQR 1.98 to 2.81] vs. median 2.35 %Inc/g [IQR 1.77 to 3.00], *P *= 0.916), while the uptake in carotid plaques was significantly higher than in renal arteries (median 2.32 %Inc/g [IQR 1.86 to 2.80] vs. median 0.44 %Inc/g [IQR 0.18 to 0.68], *P *< 0.001) (Figure 3).

### Comparison of ^18^F-NaF PET VOIs with CT Calcification VOIs

The ^18^F-NaF PET VOIs of the carotid plaques had a size of median 41 mm^3^ (IQR 20 to 74), consisting of median 6% (IQR 3 to 10) of the total plaque volume (Table 3). The binding of ^18^F-NaF in the ^18^F-NaF PET VOIs was median 4.7 (IQR 3.9 to 6.2) %Inc/g (Table 4). Overall, only a median of 35% (IQR 6 to 42) of the ^18^F-NaF PET VOI areas consisted of CT assessed calcification.

**Table 3:** Comparison of ^18^F-NaF PET VOI with CT calcification VOI

	^18^F-NaF PET VOI			CT calcification VOI		
No.	Size (mm^3^)	^18^F-NaF PET VOI of total plaque volume (%)	CT calcification volume within ^18^F-NaF PET VOI (%)	Size (mm^3^)	CT calcification VOI of total plaque volume (%)	^18^F-NaF PET volume within CT calcification VOI (%)
1	74	15	1	2	0.4	21
2	53	6	38	202	22	10
3	130	2	37	143	13	34
4	12	2	33	86	13	4
5	20	2	14	146	17	2
6	36	4	50	319	33	6
7^a^	46	9	–	–	–	–
8	71	9	43	342	41	9
9	21	2	38	156	18	5
10^b^	165	10	40	487	29	14
11	172	17	46	162	16	49
12	35	4	45	152	16	10
13	73	7	4	13	1	22
14	20	6	28	16	3	36
15	17	3	3	16	2	3
16	28	4	24	468	47	1

^18^F-NaF PET VOI was defined as ≥ 50% of maximum ^18^F-NaF uptake; CT calcification VOI was defined as Hounsfield Units ≥ 1000

*VOI*, volume of interest; *PET*, positron emission tomography; ^*18*^*F-NaF*, ^18^F- Sodium Fluoride; *CT*, computed tomography; *HU*, Hounsfield Units

^a^In this carotid plaque, no calcifications could be identified on CT scan

^b^Non-culprit carotid plaque

**Table 4:** ^18^F-NaF uptake and HU-values in PET VOI and in CT VOI

No.	^18^F-NaF uptake in PET VOI	^18^F-NaF uptake in CT VOI	Averaged HU-value PET VOI	Averaged HU-value CT VOI
1	3.6	1.9	− 295	2273
2	5.9	2.5	1367	3616
3	4.8	3.7	1293	3489
4	7.6	3.0	603	3085
5	5.1	1.5	206	2769
6	5.8	2.8	1512	3616
7^a^	2.6	–	− 361	–
8	4.6	1.9	1140	3258
9	4.0	1.9	1242	3755
10^b^	4.4	2.8	1102	3312
11	3.8	3.2	1835	3645
12	6.2	3.2	1414	3115
13	3.4	2.6	57	2465
14	7.2	5.5	699	2152
15	4.1	1.5	− 137	1885
16	6.5	2.6	568	3289

^18^F-NaF uptake was expressed as percentage uptake of total incubation dose per gram

^*18*^*F-NaF*, ^18^F- Sodium Fluoride; *PET*, positron emission tomography; *CT*, computed tomography; *HU*, hounsfield units; *VOI*, volume of interest

(%Inc/g). ^18^F-NaF PET VOI was defined as ≥ 50% of maximum ^18^F-NaF uptake; CT calcification VOI was defined as Hounsfield Units ≥ 1000

^a^In this carotid plaque, no calcifications could be identified on CT scan

^b^Non-culprit carotid plaque

The CT calcification VOI had a size of median 149 mm^3^ (IQR 16 to 290), consisting of median 16% (IQR 3 to 27) of the total plaque volume (Table 3). The overall binding of ^18^F-NaF in the CT calcification VOIs was lower than in the ^18^F-NaF PET VOI (median 2.6 [IQR 1.9 to 3.2] %Inc/g) (Table 4). Overall, a median of 10% (IQR 4 to 25) of the CT calcification VOI areas showed ^18^F-NaF uptake.

The averaged HU of the ^18^F-NaF PET VOIs (median 901 HU [IQR 94 to 1349]) was lower than that of the CT calcification VOIs (median 3258 HU [IQR 2465 to 3616]), as these ^18^F-NaF PET VOIs consist only partially of CT assessed calcification (Table 4). In renal arteries no CT calcification VOI could be detected; median HU of total renal artery volume was -470 HU (IQR − 530 to − 390).

**Figure 4:**
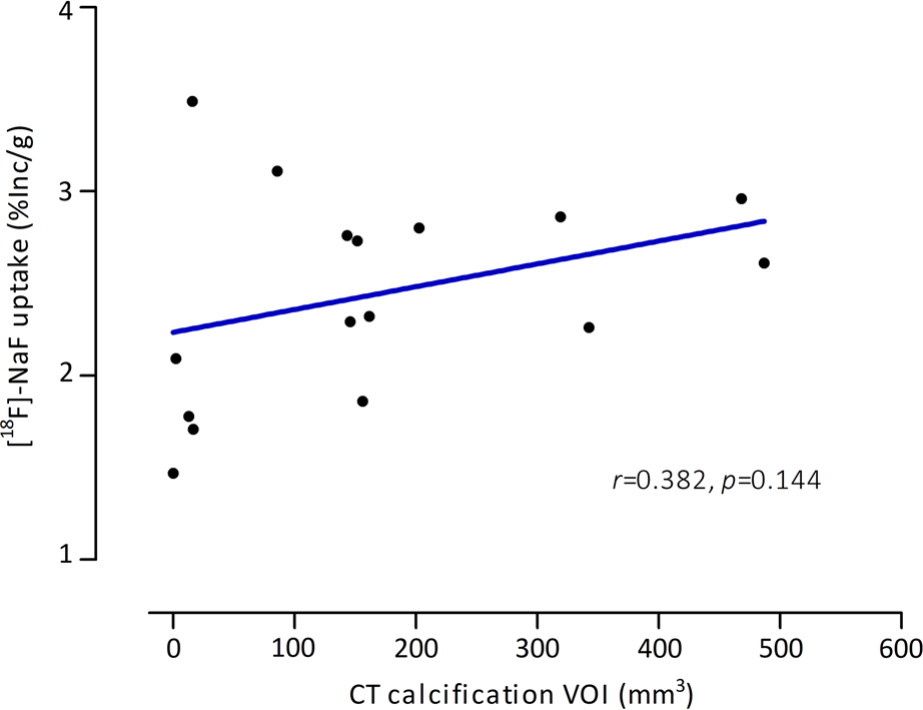
Relation between PET assessed ^18^F-NaF uptake and CT assessed calcification in human carotid plaques. CT assessed calcification VOI was defined as HU-value ≥ 1000. Data is expressed as percentage ^18^F-NaF uptake of the total incubation dose per gram of tissue (% Inc/g). Spearman Correlation was used to assess the relation between ^18^F-NaF uptake and CT assessed calcification

No significant association was found between ^18^F-NaF uptake of the plaque (%Inc/g) and the CT calcification VOI (*r *= 0.382, *P *= 0.144), although ^18^F-NaF uptake seems to increase when CT calcification VOI increases in most plaques (Figure 4).

### Histological Staining

The von Kossa and alizarin red stainings showed calcification deposits in the two selected ^18^F-NaF-positive, CT negative segments of the carotid plaques. The pattern of ^18^F-NaF uptake on PET images matched with the area of histologically proven calcification (Online Resource 2, Figure 1). Areas with negligible ^18^F-NaF uptake did not show any histological evidence for calcification. As expected, no calcifications were identified in the segments of the renal arteries (Online Resource 3, Figure 2).

The five culprit and three non-culprit plaques segments showed all at least one characteristic of vulnerability. Macrophage infiltration was clearly visible in all segments, as was the presence of intraplaque microvessels, without a visual difference in culprit or non-culprit segments. Two culprit plaques and one non-culprit plaque showed a clear thrombus indicative of intraplaque hemorrhage. One non-culprit plaque showed a very thin fibrous cap (Online Resource 4, Figure 3).

## Discussion

The present study investigated in vitro microPET assessed ^18^F-NaF uptake, in culprit and non-culprit human carotid plaques. We hypothesized that ^18^F-NaF uptake in culprit plaques was higher than in non-culprit carotid plaques, based on previous results in coronary plaques, and the concept of microcalcification and plaque rupture.^15^,^22^ Our results, however, demonstrate comparable ^18^F-NaF uptake in culprit and non-culprit carotid plaques. Interestingly, we found that ^18^F-NaF uptake was present in regions without evidence of calcification on CT scan. Furthermore, most of the CT calcification VOIs had low ^18^F-NaF uptake, confirming that both techniques represent a different stage of calcification.^14^,^23^,^32^

Recently, Vesey et al. showed that in vivo ^18^F-NaF uptake was higher in culprit carotid artery stenosis than in the contralateral non-culprit stenosis in 18 patients with recent CVA (log_10_ standardized uptake value, mean 0.29 ± 0.10 vs 0.23 ± 0.11 respectively, *P < *0.001).^23^ These findings are consistent with the results of a clinical study of Quirce et al., in nine patients, where ^18^F-NaF uptake reported as mean target-to-background ratio was higher in culprit plaques (2.12 ± 0.44) than in contralateral non-culprit plaques (1.85 ± 0.46, *P *= 0.220).^24^ Age and sex distribution were comparable between these two studies and our study. Only Vesey et al. provided information about the cardiovascular history and other cardiovascular risk factors of the included patients. The prevalence of smoking and diabetes mellitus was similar. Furthermore, more than 50% of the patients had other manifestations of atherosclerosis, as in our study. Since the included patients in both studies were comparable, the remaining question is how these contradictive results can be explained.

First, although Vesey et al. did find a significant difference between ^18^F-NaF uptake in culprit and contralateral non-culprit plaques, the differences were small and a substantial overlap between the uptake values in both groups was present, as was in the study of Quirce et al. Furthermore, the ^18^F-NaF uptake between patients scheduled for CEA and control patients differed to a larger extent (delta 0.17 SUV_mean_) than the difference between culprit and non-culprit uptake (delta 0.07 SUV_mean_).^23^ Therefore, we believe that no absolute cut off value for the diagnosis of culprit plaques based on ^18^F-NaF uptake can be determined, only when compared with control patients there is a relevant difference. This is in line with the results of our study. Although the sample size is small, our data show that ^18^F-NaF uptake between culprit and non-culprit carotid plaques was comparable while the uptake in plaques was significantly higher than in control renal arteries.

Second, there may be differences in the degree of stenosis of the carotid arteries between patients in the aforementioned studies and our patients. Vesey et al. found that ^18^F-NaF uptake was related to the degree of stenosis on CT, but they did not report the stenosis degree in the separate groups, neither did Quirce et al. In our study, the stenosis degree in both, non-culprit and the culprit plaques, was high and all plaques showed high-risk features based on histology. This could explain the similar ^18^F-NaF uptake.

In contrast, Joshi et al. did find a difference between culprit and non-culprit *coronary* plaques of patients with myocardial infarction.^22^ This might be the consequence of local differences in the mechanical forces exerted to the artery wall and the endothelium of the coronary vascular bed, causing local differences in plaque initiation and progression.^33^ In contrast to the coronary arteries, both carotid arteries are exposed to similar blood flow patterns and mechanical stress. This might result in the same pattern of plaque development and progression.

^18^F-NaF activity was increased in areas without calcification on CT and most of the CT calcification VOI showed minimal ^18^F-NaF uptake. This supports the idea that microcalcification, as visualized with ^18^F-NaF PET, and established calcification visualized on CT may reflect different stages of the calcification processes in atherosclerotic plaques.^23^,^27^,^34^

Established calcification is a well-known marker of total plaque burden and is strongly associated with the risk for cardiovascular events.^35^ However, the amount of established calcification, as detected by CT, only provides information about the processes in the past and not about the actual biological activity of the plaque.^36^ Moreover, larger and denser areas of calcification may even stabilize the plaque.^37^ This has, for example, been suggested by Shalaan et al., who found a higher CT assessed calcification volume in non-culprit than in culprit carotid plaques.^38^ The average percentage of plaque volume that was calcified was comparable with our study. Unfortunately, in our study CT assessed calcification volume could not be compared between culprit and non-culprit plaques, due to limited availability of CT images in the non-culprit group (n = 1) because of image reconstruction failures.

Another important example can be derived from the work of Puri et al., who performed a post-hoc patient-level analysis of 8 prospective trials in which coronary atheroma was measured with intravascular ultrasound. They showed that although statins had a clear plaque-regressive effect, they also promoted coronary atheroma calcification, indicating stabilization.^39^

In this study increased ^18^F-NaF uptake was related to the calcium volume on CT in the majority of the carotid plaques. This is probably caused by binding of ^18^F-NaF at only the surface of the calcifications.^14^ The binding of fluoride to hydroxyapatite is based on ion exchange, rather than incorporation by active transport. This probably also explains why ^18^F-NaF uptake can still be found in vitro.

However, in our study a few plaques with low calcium volume had a high ^18^F-NaF uptake and vice versa. This suggests ^18^F-NaF accumulation in areas without any evidence of calcification, or at least no calcification with a size above the detection limit of the microCT scan.^14^ The presence of calcifications smaller than the CT detection limit, i.e. microcalcifications was indeed confirmed by histological staining of ^18^F-NaF positive and CT negative segements. ^18^F-NaF probably binds to the relatively large surface of microcalcifications , causing an intense signal on PET images.^14^

These observations indicate that ^18^F-NaF imaging can detect biologically active plaques, before they can be visualized on CT. This implies that ^18^F-NaF imaging may be useful in evaluating disease progression, as was further shown in patients with aortic stenosis, where baseline ^18^F-NaF uptake correlated well with the calcium progression after 1 year.^40^ Especially, ^18^F-NaF uptake in areas without established calcification on CT was the best predictor of calcium progression.

Furthermore, Derlin et al. found a positive correlation between ^18^F-NaF uptake in the carotid arteries and, age and various cardiovascular risk factors in 269 patients with no history of stroke.^32^ Derlin et al. included a heterogeneous population, consisting of patients with low or minimal cardiovascular risk as well. In contrast, we included only patients with already a history of cardiovascular disease and, therefore, at a high-risk for a cardiovascular event. These findings further highlight the possibility of ^18^F-NaF imaging to identify patients at high-risk for cardiovascular disease in a low-risk population. In addition, the finding that ^18^F-NaF uptake in carotid plaques exceeded that of controls (renal arteries) and no calcification was visible in renal arteries on microCT or with histological staining adds evidence to the hypothesis that the presence of microcalcification identified by ^18^F-NaF is a feature of atherosclerosis. The relation between the extent of ^18^F-NaF uptake in bilateral carotid plaques and features of vulnerability needs to be further investigated.

Strengths of our study are the inclusion of a control group, and the scanning of calcium phantoms in order to accurately determine the calcium threshold. Furthermore, by comparing calcification identified by ^18^F-NaF PET imaging with calcification visualized on microCT, this study adds knowledge to the relatively new field of ^18^F-NaF imaging in atherosclerosis. Our study has some limitations. First, it should be considered that the number of plaques, especially non-culprit plaques, is small and a type II statistical error might be introduced. However, given the similar distribution of ^18^F-NaF uptake in both groups, a high number of plaques would need to be recruited to find a statistical significant difference if present. We believe that small differences in uptake will not have any clinical implication.

Second, the tracer uptake could be underestimated due to partial volume effects, as with every imaging study. Third, CT images of 16 plaques (one non-culprit) out of 23 were available for analysis due to practical and technical issues. Fourth, we did not assess the plaque morphology of the right and left side in the same patient. Culprit and non-culprit plaques were derived from different patients.

## New Knowledge Gained

We have demonstrated that the calcification patterns on ^18^F-NaF PET images and CT images are different. Clearly, ^18^F-NaF PET visualizes a different stage of the calcification process than CT. ^18^F-NaF uptake in carotid plaques exceeded the uptake in non-calcified renal arteries, but was comparable between culprit and non-culprit carotid plaques, probably due to the advanced nature of atherosclerotic disease in our patients.

## Conclusion

We conclude that ^18^F-NaF has the potential to identify carotid plaques with active calcification. Further prospective studies on ^18^F-NaF uptake and symptomatology are required to assess the predictive and diagnostic value of ^18^F-NaF imaging in patients with early stage atherosclerosis.

## Electronic supplementary material

Below is the link to the electronic supplementary material.

Online Resource 1 (DOCX 20 kb)

Online Resource 2 (JPEG 660 kb)

Online Resource 3 (JPEG 109 kb)

Online Resource 4 (JPEG 3228 kb)

Online Resource 5 (PPTX 463 kb)

## Abbreviations

^18^F-NaF: ^18^F- sodium fluoride
PET: Positron emission tomography
CT: Computed tomography
CEA: Carotid endarterectomy
PBS: Phosphate buffered saline
QMA: Quarternary methyl ammonium
VOI: Volume of interest
HU: Hounsfield Unit
MSB: Martinus Scarlet Blue
IQR: Interquartile range
BMI: Body mass index
