# Supplementary material for: 18F-sodium fluoride positron emission tomography assessed microcalcifications in culprit and non-culprit human carotid plaques
Source: J Nucl Cardiol. 2018 Jun 25;26(4):1064–75. doi: 10.1007/s12350-018-1325-5 (PMC6660502; doi:10.1007/s12350-018-1325-5)
Supplement: Supplementary file 1 — Online Resource 1 (DOCX 20 kb) [file 12350_2018_1325_MOESM1_ESM.docx]

**Online Resource 1 (supplemental)**

**Staining procedures**

***General procedures***

Carotid plaques and renal artery segments were cut in sections of 5 µm, deparaffinised with xylene, and rehydrated with ethanol and demineralized water. The two renal arteries with the highest ^18^F-NaF uptake and two ^18^F-NaF positive, but CT negative carotid plaques segments were stained for calcification. Furthermore, five culprit and three non-culprit plaques were stained for the presence of CD68- expressing cells (macrophages) and CD34- expressing cells (endothelial cells). The presence of intraplaque thrombus and collagenous fibrous cap was evaluated using standardized Martinus, Scarlet and Blue (MSB) staining.

***Calcification staining***

Calcifications in carotid plaques and renal artery segments were identified with Alizarin Red staining and von Kossa staining. In brief, sections were incubated in 2% Alizarin Red for five minutes at room temperature. After incubation, sections were dipped 20 times in 1:1 acetone:xylene, followed by 100% xylene. Then, the sections were rinsed with ethanol and dried.

For the von Kossa staining, the sections were incubated in 1% silver nitrate solution and exposed to sunlight for 30 minutes. Then, sections were rinsed with demineralized water, and 3% thiosulfate was added for five minutes. After the sections were rinsed again, Nuclear Fast Red counterstain was added for three minutes, after which the sections were washed with ethanol and dried.

***Staining of macrophages and microvessels***

For the staining of macrophages, segments were incubated with CD68 (mouse IgG_3_ κ monoclonal, Dako, clone KP-1). For staining of microvessels CD34 was used (mouse IgG1 κ monoclonal, Dako, clone QBEnd-10, code number M7165). After an incubation time of one hour at room temperature, the segments were incubated with horseradisch peroxidase (HPR) labeled secondary antibodies. Then, 3,3'-diaminobenzidine (DAB) chromogenic staining was used to visualize the antibodies. Last, segments were counterstained with hematoxylin.

***Analysis of staining***

Digital images of the stained sections were made using the NanoZoomer Digital Pathology Scanner (Hamamatsu Photonics K.K., Japan). The images were inspected using pathology viewing software (Aperio ePathology, LeicaBiosystems, the Netherlands) and were visually reviewed by one observer with broad experience in vascular pathology.
