# Supplementary figures and images for: 18F-sodium fluoride positron emission tomography assessed microcalcifications in culprit and non-culprit human carotid plaques
Source: J Nucl Cardiol. 2018 Jun 25;26(4):1064–75. doi: 10.1007/s12350-018-1325-5 (PMC6660502; doi:10.1007/s12350-018-1325-5)

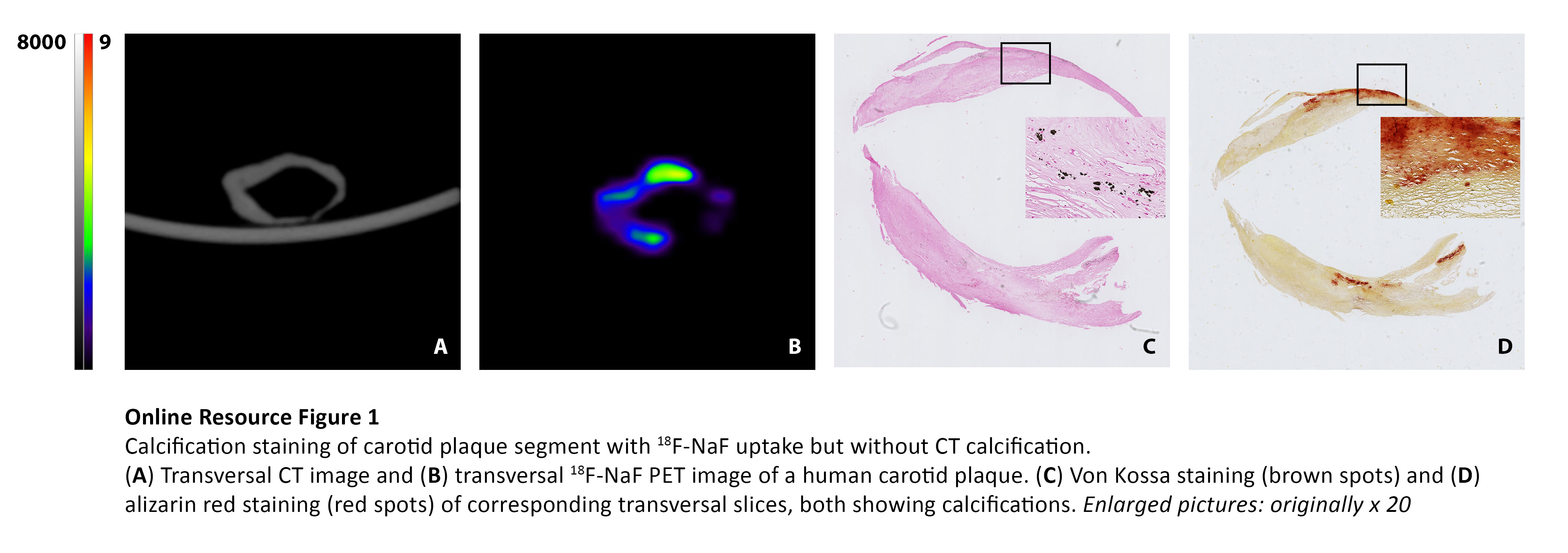

Supplement: Supplementary file 2 — Online Resource 2 (JPEG 660 kb) [file 12350_2018_1325_MOESM2_ESM.jpg]

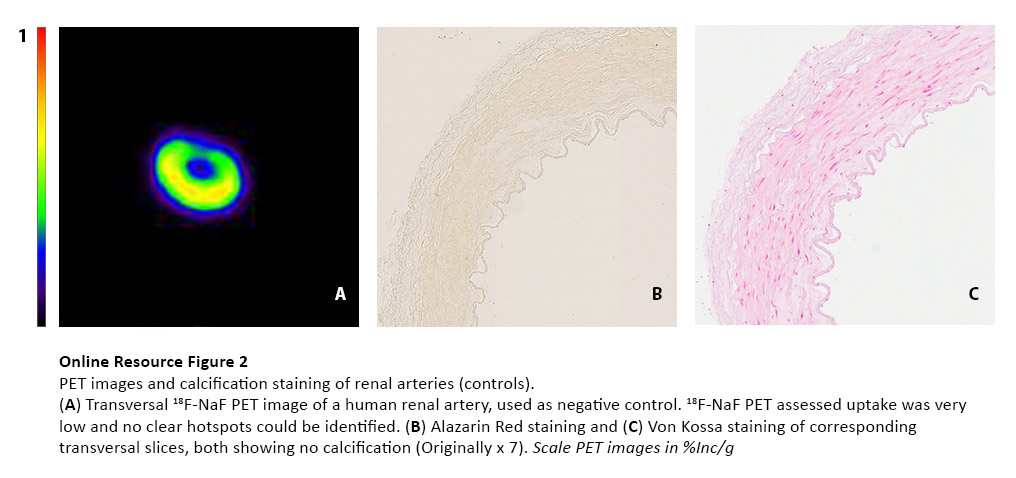

Supplement: Supplementary file 3 — Online Resource 3 (JPEG 109 kb) [file 12350_2018_1325_MOESM3_ESM.jpg]

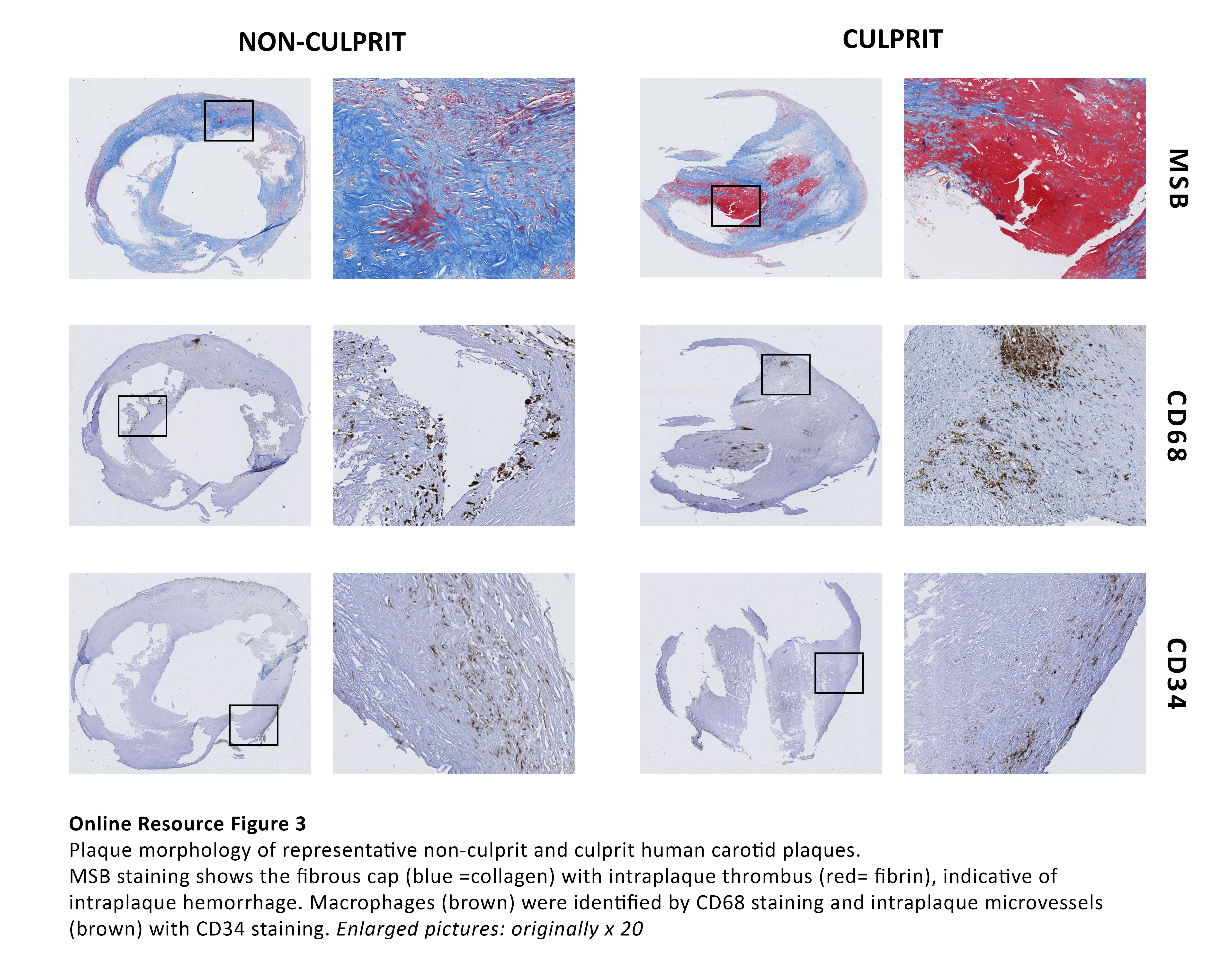

Supplement: Supplementary file 4 — Online Resource 4 (JPEG 3228 kb) [file 12350_2018_1325_MOESM4_ESM.jpg]
