# Supplementary material for: 18F-sodium fluoride positron emission tomography assessed microcalcifications in culprit and non-culprit human carotid plaques
Source: J Nucl Cardiol. 2018 Jun 25;26(4):1064–75. doi: 10.1007/s12350-018-1325-5 (PMC6660502; doi:10.1007/s12350-018-1325-5)
Supplement: Supplementary file 5 — Online Resource 5 (PPTX 463 kb) [file 12350_2018_1325_MOESM5_ESM.pptx]

## Slide 1
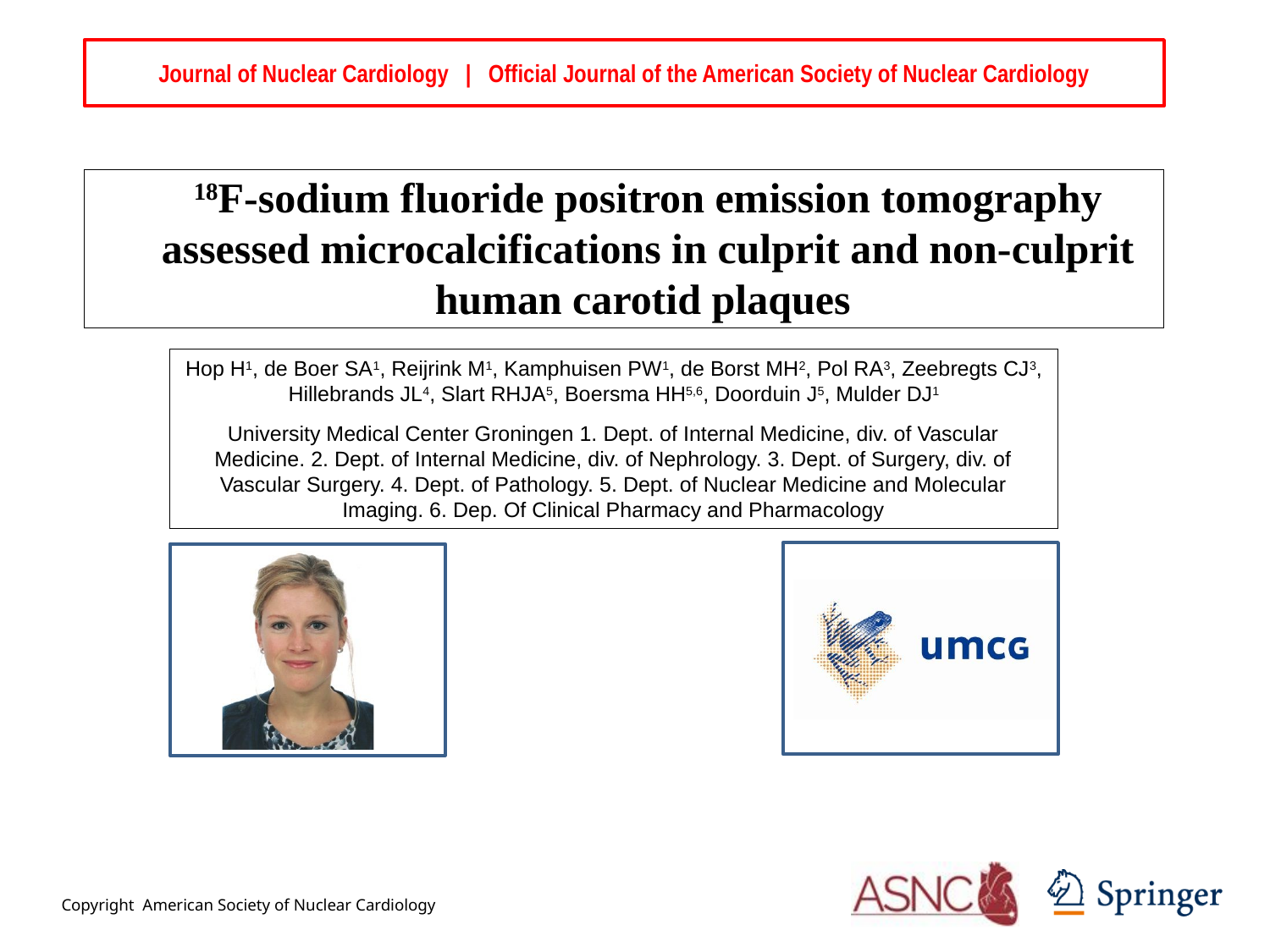

Journal of Nuclear Cardiology | Official Journal of the American Society of Nuclear Cardiology
# 18F-sodium fluoride positron emission tomography assessed microcalcifications in culprit and non-culprit human carotid plaques
Hop H1, de Boer SA1, Reijrink M1, Kamphuisen PW1, de Borst MH2, Pol RA3, Zeebregts CJ3, Hillebrands JL4, Slart RHJA5, Boersma HH5,6, Doorduin J5, Mulder DJ1
University Medical Center Groningen 1. Dept. of Internal Medicine, div. of Vascular Medicine. 2. Dept. of Internal Medicine, div. of Nephrology. 3. Dept. of Surgery, div. of Vascular Surgery. 4. Dept. of Pathology. 5. Dept. of Nuclear Medicine and Molecular Imaging. 6. Dep. Of Clinical Pharmacy and Pharmacology
Copyright American Society of Nuclear Cardiology

## Slide 2
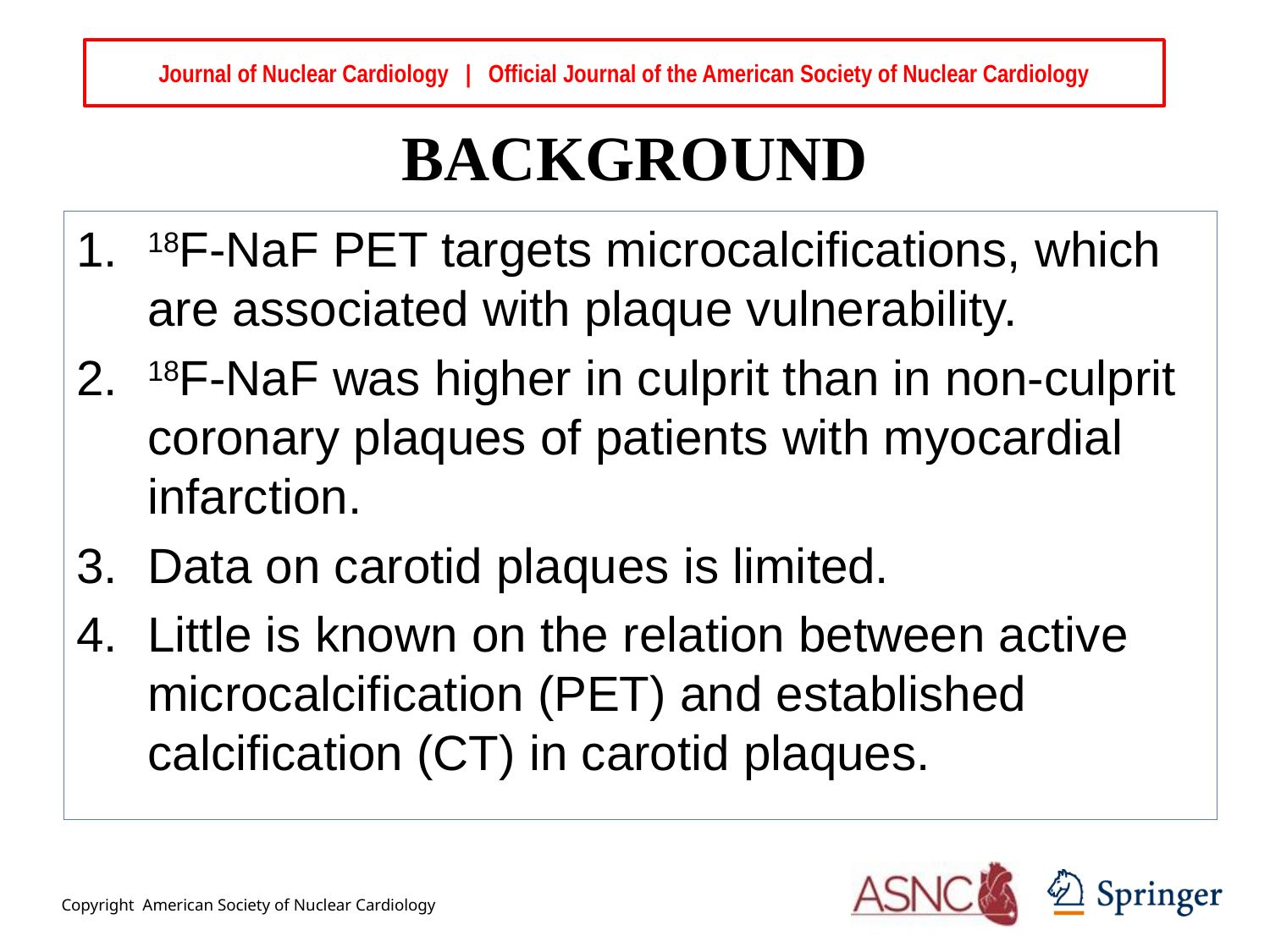

Journal of Nuclear Cardiology | Official Journal of the American Society of Nuclear Cardiology
# BACKGROUND
18F-NaF PET targets microcalcifications, which are associated with plaque vulnerability.
18F-NaF was higher in culprit than in non-culprit coronary plaques of patients with myocardial infarction.
Data on carotid plaques is limited.
Little is known on the relation between active microcalcification (PET) and established calcification (CT) in carotid plaques.
Copyright American Society of Nuclear Cardiology

## Slide 3
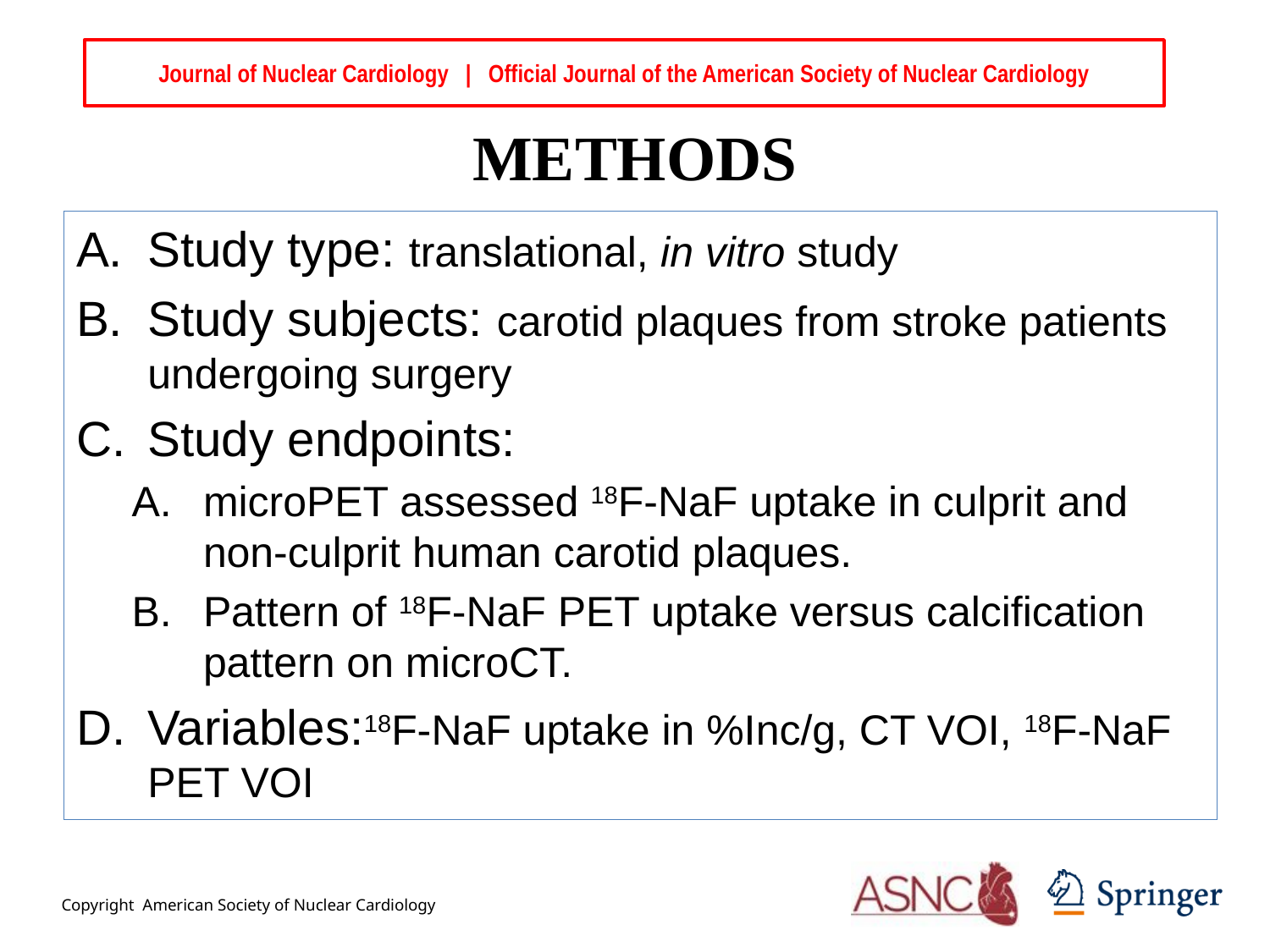

Journal of Nuclear Cardiology | Official Journal of the American Society of Nuclear Cardiology
# METHODS
Study type: translational, in vitro study
Study subjects: carotid plaques from stroke patients undergoing surgery
Study endpoints:
microPET assessed 18F-NaF uptake in culprit and non-culprit human carotid plaques.
Pattern of 18F-NaF PET uptake versus calcification pattern on microCT.
Variables:18F-NaF uptake in %Inc/g, CT VOI, 18F-NaF PET VOI
Copyright American Society of Nuclear Cardiology

## Slide 4
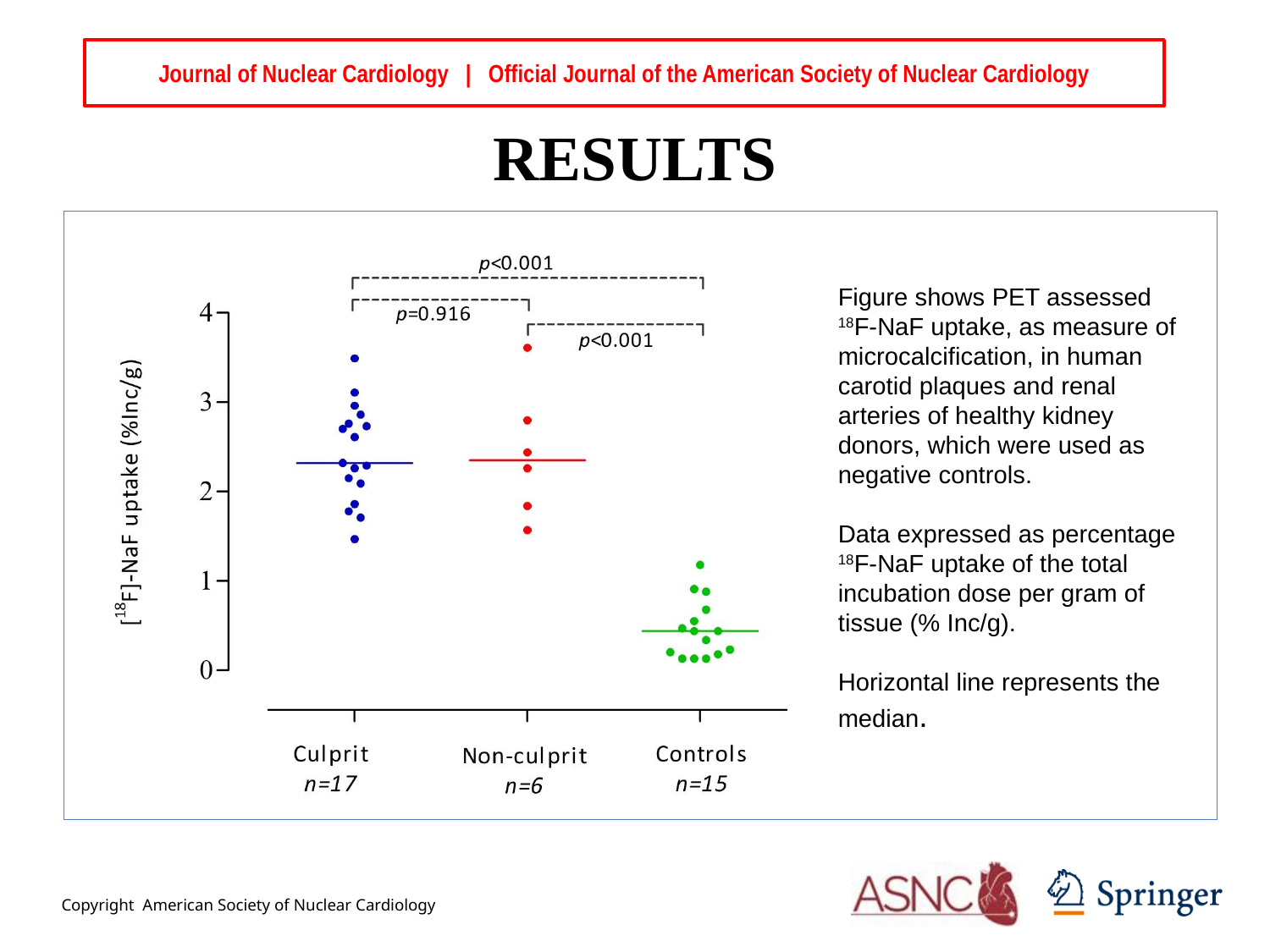

Journal of Nuclear Cardiology | Official Journal of the American Society of Nuclear Cardiology
# RESULTS
H
Figure shows PET assessed 18F-NaF uptake, as measure of microcalcification, in human carotid plaques and renal arteries of healthy kidney donors, which were used as negative controls.
Data expressed as percentage 18F-NaF uptake of the total incubation dose per gram of tissue (% Inc/g).
Horizontal line represents the median.
Copyright American Society of Nuclear Cardiology

## Slide 5
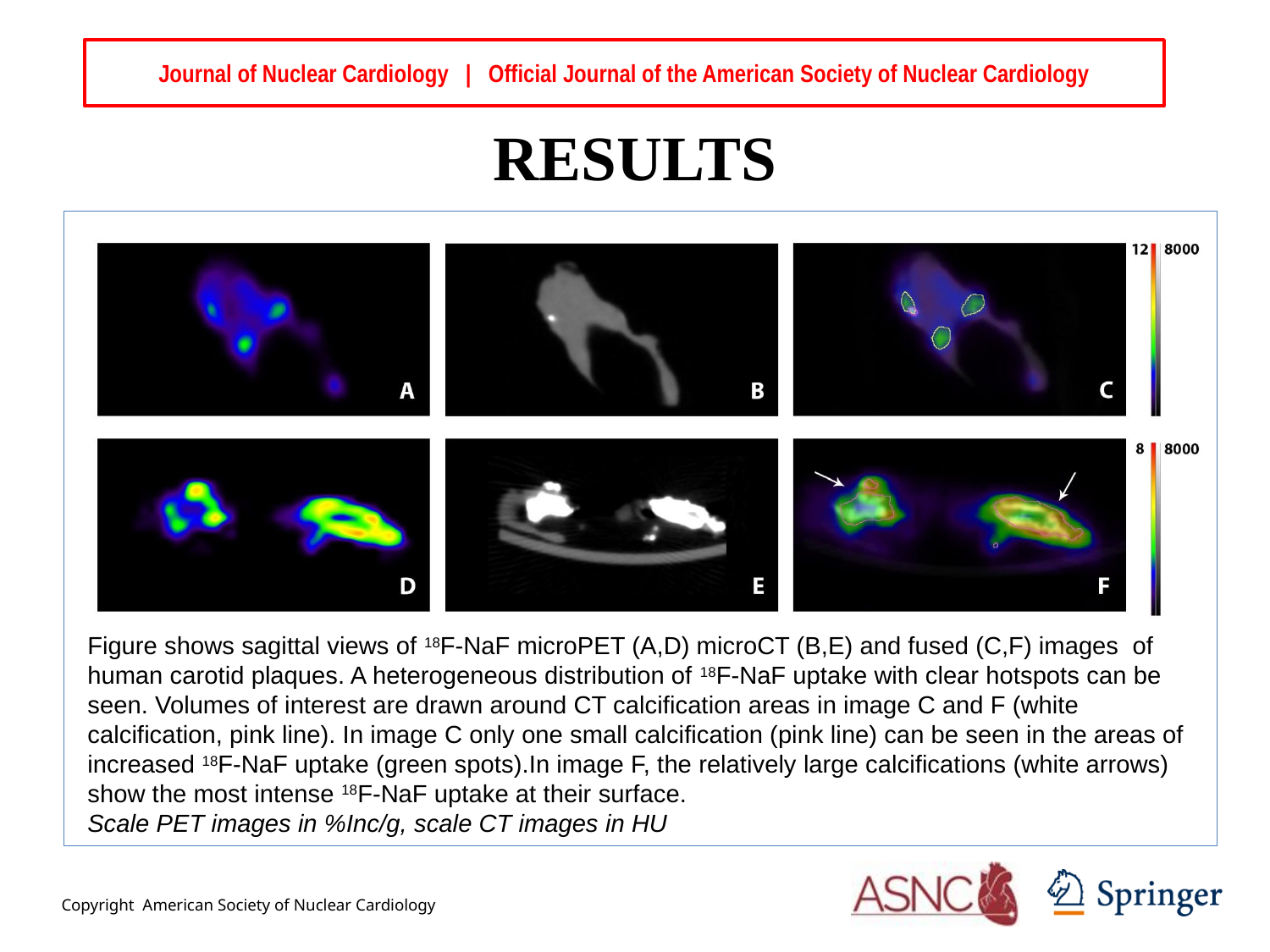

Journal of Nuclear Cardiology | Official Journal of the American Society of Nuclear Cardiology
# RESULTS
Figure shows sagittal views of 18F-NaF microPET (A,D) microCT (B,E) and fused (C,F) images of human carotid plaques. A heterogeneous distribution of 18F-NaF uptake with clear hotspots can be seen. Volumes of interest are drawn around CT calcification areas in image C and F (white calcification, pink line). In image C only one small calcification (pink line) can be seen in the areas of increased 18F-NaF uptake (green spots).In image F, the relatively large calcifications (white arrows) show the most intense 18F-NaF uptake at their surface.
Scale PET images in %Inc/g, scale CT images in HU
Copyright American Society of Nuclear Cardiology

## Slide 6
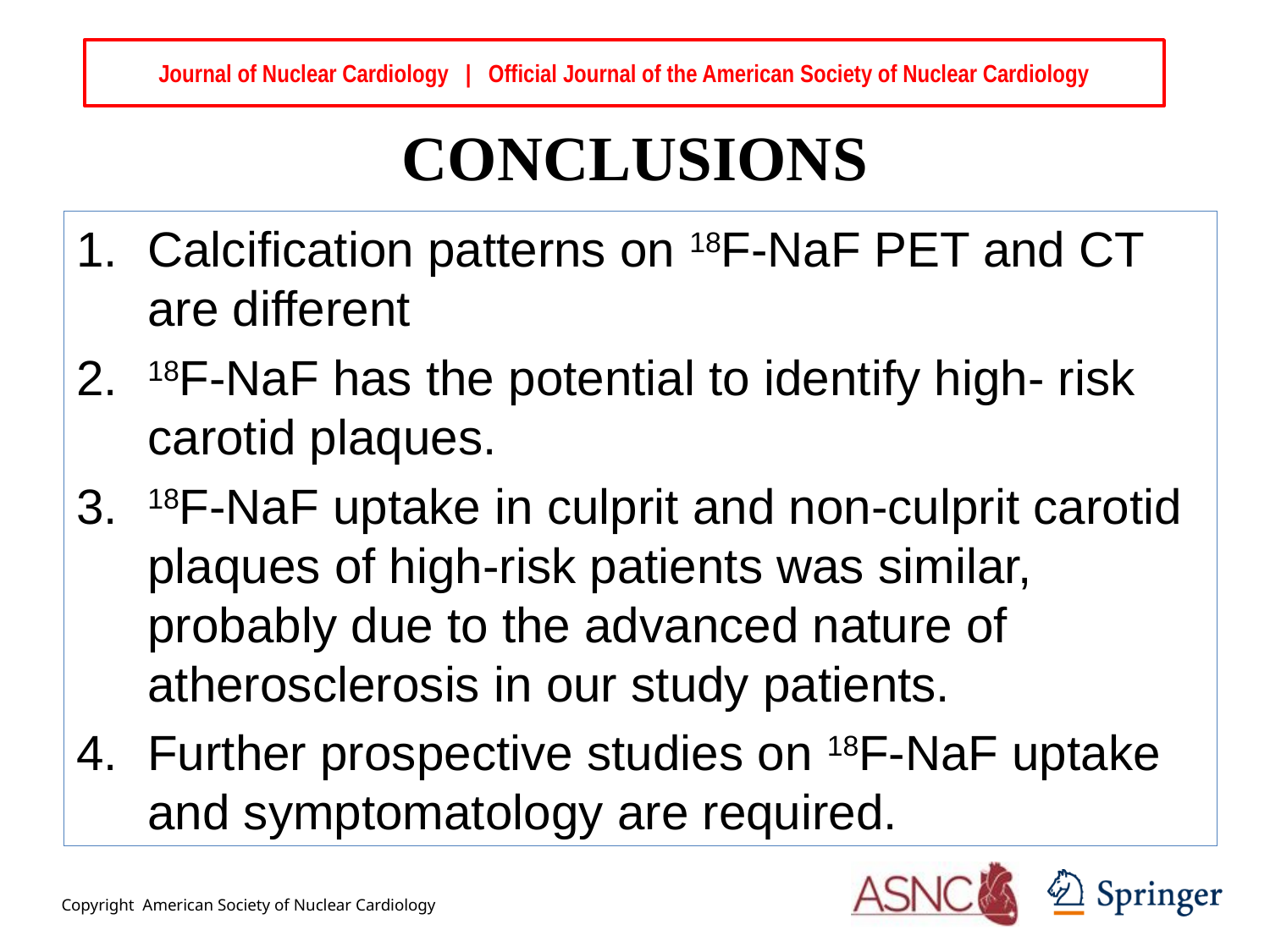

Journal of Nuclear Cardiology | Official Journal of the American Society of Nuclear Cardiology
# CONCLUSIONS
Calcification patterns on 18F-NaF PET and CT are different
18F-NaF has the potential to identify high- risk carotid plaques.
18F-NaF uptake in culprit and non-culprit carotid plaques of high-risk patients was similar, probably due to the advanced nature of atherosclerosis in our study patients.
Further prospective studies on 18F-NaF uptake and symptomatology are required.
Copyright American Society of Nuclear Cardiology
